# Analysis and Acoustic Event Classification of Environmental Data Collected in a Citizen Science Project

**DOI:** 10.3390/ijerph20043683

**Published:** 2023-02-19

**Authors:** Daniel Bonet-Solà, Ester Vidaña-Vila, Rosa Ma Alsina-Pagès

**Affiliations:** Human Environment Research (HER), La Salle—Universitat Ramon Llull, Sant Joan de La Salle, 42, 08022 Barcelona, Spain

**Keywords:** citizen science, acoustic event detection, noise annoyance, convolutional neural networks

## Abstract

Citizen science can serve as a tool to obtain information about changes in the soundscape. One of the challenges of citizen science projects is the processing of data gathered by the citizens, to obtain conclusions. As part of the project *Sons al Balcó*, authors aim to study the soundscape in Catalonia during the lockdown due to the COVID-19 pandemic and afterwards and design a tool to automatically detect sound events as a first step to assess the quality of the soundscape. This paper details and compares the acoustic samples of the two collecting campaigns of the *Sons al Balcó* project. While the 2020 campaign obtained 365 videos, the 2021 campaign obtained 237. Later, a convolutional neural network is trained to automatically detect and classify acoustic events even if they occur simultaneously. Event based macro F1-score tops 50% for both campaigns for the most prevalent noise sources. However, results suggest that not all the categories are equally detected: the percentage of prevalence of an event in the dataset and its foregound-to-background ratio play a decisive role.

## 1. Introduction

Every year, environmental noise causes 48,000 new cases of ischaemic heart disease and more than 12,000 deaths in Europe [1]. Recent studies by the World Health Organisation (WHO) present recommendations regarding the equivalent levels of noise that should not be exceeded in different environments (road traffic, railway noise, and leisure noise, among others). Also, more than 6.5 million people suffer from chronic high sleep disturbance due to noise [2]. The numbers increase to more than 22 million people when chronic high annoyance is included.

Several studies show that environmental noise causes sleep disorders with awakenings that have negative effects on health and quality of life [3,4]. Other detrimental effects of noise pollution on health are hypertension ischemic heart diseases [5,6], diastolic blood pressure [7] and psychological stress [8]. Environmental noise has also been associated with learning impairment [9,10], reduction of working performance [11,12] and general annoyance [13], all of which impact negatively on the well-being of the general population.

The first step in tackling the deteriorated soundscape existing in many cities across the world is to further analyze its current state through a combination of objective data collection and the subjective perceptions of citizens. This information can then be used to develop tools for evaluating the quality of the soundscape.

Last 2020, and especially during March, April and May, the soundscape of our cities changed drastically [14,15] due to the COVID-19 pandemic. All the sounds [16] associated with daily activity nearly disappeared, especially during the strictest closing weeks. Noise from road traffic [17], railroads, airports, or even leisure activities showed a significant decrease in the majority of cities studied [18,19]. *Sons al Balcó* is a citizen science project, initially launched to analyze the impact of the lockdown on the soundscape of Catalonia [20]. At that moment, and in response to the lock down measures, other similar projects were launched worldwide: in the United Kingdom [21], Italy [22,23,24], in New York City [25] and even worldwide [26]. These projects had the goal of registering the exceptional soundscape conditions in all the cities.

The starting hypothesis of the *Sons al Balcó* project was that the reduction in outdoor noise caused by the lockdown, could be associated with greater well-being of people. The project proposed to citizens a double contribution: on the one hand, the recording of short videos with the mobile phone (around 30 s), that should be uploaded to the cloud, and on the other hand, answering a questionnaire about the changes in the perception of the soundscape during the lockdown. The questionnaires focused on how citizens had experienced the changes in their acoustic environment, asking them for pleasant changes but also for annoyance factors. The ultimate goal was to evaluate these responses in conjunction with the objective data obtained from the videos.

*Sons al Balcó* conducted two collecting campaigns across Catalonia, among other activities. One during the COVID-19 lockdown, in 2020, and another just one year later, in 2021. The target was to observe the changes in the soundscape in those two years. This gave the team the opportunity to compare the samples collected during the lockdown with the possibility of carrying out a more exhaustive analysis of those data using the videos collected post-lockdown.

As part of the growing concern over the environmental noise in many urban areas, the broader scope of the *Sons al Balcó* project was to engage citizens, schools and administration to (1) draw the soundscape map of Catalonia by collecting objective and subjective data, (2) create social and environmental impact, (3) raise awareness about noise pollution and (4) design tools to enhance citizens’ knowledge and empower them.

Both campaigns were advertised on social media, so all the citizens living in Catalonia could participate in the study. For a video to be considered valid for the study, it should satisfy three requirements:

1. It should contain data recorded from a balcony or window.
2. It should be recorded in Catalonia.
3. It should not contain human faces.

Prior to their publication, all the videos were manually analyzed to guarantee that the three requirements specified above were satisfied. After that, all the valid videos were manually analysed and labelled according to a taxonomy. As it was common to find overlapping acoustic events, the audio files were labelled considering polyphony (multiple labels occurring at the same time). A total of 365 videos were obtained during the 2020 (lockdown) campaign, and other 237 were collected in the 2021 post-lockdown campaign.

In this specific context, this paper focuses on presenting and comparing the datasets obtained during the 2020 and 2021 campaigns. This work also explains the design of a tool able to perform automatic sound event classification in them. More specifically, this singular work pursues three goals: Firstly, to explain and compare these two collaboratively obtained polyphonic (also referred to as multilabel) datasets collected during the COVID-19 lockdown and the year after across Catalonia. These datasets are different from most of the datasets used for polyphonic classification in the literature as the audio clips come from different scenarios and sound sensors. Mainly, all the audio clips were recorded by hundreds of participants in a citizen science project using smartphones or other non-professional devices. The datasets include informative metadata, such as the location of the recordings and have been annotated using a hierarchical taxonomy for describing the sound elements. Moreover, the data labels also include the foreground or background placement of the sound. In contrast to monophonic datasets, where a single event is active simultaneously, in polyphonic datasets (such as the ones presented in this paper) several labelled acoustic events occur simultaneously. In addition to that, one dataset was collected in the middle of severe activity and mobility restrictions while the other was collected in a normalized context free of the pandemic regulations, offering the opportunity to compare both scenarios.

Secondly, to design a system to automatically classify the sound events in those datasets. The authors also aim to study the viability of training a model with the data obtained in past campaigns in order to automatically classify sounds of future campaigns, even when the environmental context may change. As not all noise sources are equally annoying, it is important to have a tool to automatically detect the specific predominant sounds existing in an urban location in order to better assess the welfare or level of annoyance for its inhabitants.

The final contribution of the present study is to analyze the effects of two features related to the dataset composition in the classification process. Specifically, the authors have searched for possible correlations between the prevalence of each sound class in the dataset and the foreground-to-background ratio and the classification performance.

The rest of the paper is organized as follows. In Section 2, there is a compilation of the related work published to this date. Section 3, specifies the methodology and setting of the classification algorithm. Section 4 presents a full description of the datasets collected during the past *Sons al Balcó* campaigns and the results of the experiments conducted. In Section 5 results are interpreted and placed in context. Finally, Section 6 offers the main conclusions and future lines of work.

## 2. Related Work

This section covers the most relevant works in the literature related to (1) polyphonic datasets, (2) usage of Gammatone Cepstral Coefficients (GTCC) and Convolutional Neural Networks (CNN) for sound events detection and (3) approaches of multilabel classification applied to real-world urban scenarios.

### 2.1. Datasets for Sound Events Detection and Classification

Most of the datasets presented in the literature for sound research are monophonic. Some examples include UrbanSound [27] with over 18 h of tagged sound events, *freefield1010* [28] with 7690 annotated audio files containing close to 18,000 sound events obtained from Freesound (https://freesound.org, accessed on 14 February 2023), DARES-G1 [29] containing two hours of annotated real-world sound events recorded in Groningen or ESC [30] with a compilation of environmental sounds comprising up to 50 classes.

Even though there are quite a few multilabel datasets available for different kinds of data, such as images or text, multilabel datasets for polyphonic sound classification are scarce in comparison. However, they are becoming more popular thanks to the recent citizen science projects and acoustic events detection and classification challenges. There are two main approaches for creating such datasets. On the one hand, some datasets have been synthesized from isolated sounds, usually taken from existent single-label datasets. For example, the USM-SED dataset generated 20,000 polyphonic soundscapes mixing sounds from the FSD50K dataset [31]. On the other hand, some datasets have been compiled through a set of recordings, usually made by a single user or network such as SONYC-UST [32] consisting of 3068 recordings from an acoustic sensor network deployed in New York City including 23 different sound classes tagged by volunteers or SINGA:PURA [33] with a total of 18.2 h of audio data recorded through a wireless acoustic sensor network deployed in the city of Singapore. Both SONYC-UST and SINGA:PURA tagged the sound events using a hierarchical taxonomy. It is also worth mentioning Audio Set [34], a dataset collected by Google and consisting of almost two million 10 s excerpts obtained from YouTube videos and manually annotated by different human labelers using a hierarchical taxonomy of 632 audio classes. Subsets of Audio Set, such as DCASE2017, have also been assembled almost on a yearly basis to perform polyphonic sound event detection as part of the annual DCASE challenge [35]. Even though these datasets are very large, they are weakly labeled.

### 2.2. Gtcc and CNN for Audio Classification

GTCC [36] have been successfully used for audio classification. Previous work from the authors of this paper showed that they performed better than other feature extraction methods such as Mel Frequency Cepstrum Coefficients (MFCC) or Narrow Band Autocorrelation Features (NB-ACF) in classifying five diverse acoustic environments [37]. The aforementioned work classified different datasets using GTCC with Gaussian Mixture Model (GMM), *k*-Nearest Neighbors (*k*NN) and a multilayer perceptron.

In the DYNAMAP road traffic noise mapping project, GTCC were also used with *k*NN and Fisher’s Linear Discriminant (FLD) for the detection of anomalous noise events [38]. In [39], a combination of GTCC and Hidden Markov Model (HMM) along with other state-of-the-art methods were used to classify up to 50 classes from a sound scene database. In a different scenario where the target was to classify bird sounds, in [40], GTCC coupled with a Support Vector Machine (SVM) also outperformed MFCC and Linear Prediction Cepstrum Coefficients (LPCC). More recently, GTCC have also been used on a deep-learning framework consisting of a Bidirectional Long Short-Term Memory (BiLSTM) to classify respiratory sounds [41].

CNNs [42] are gaining traction as one of the preferred choices for deep learning nowadays. They have been used for audio classification on several environments, for example for bird species identification. Kahl et al. [43] used a CNN to identify bird species based on a dataset with 36,496 recordings, achieving a mean average precision of 0.605, while Koh et al [44] classified 659 bird species from 50,000 recordings also using a CNN. Shifting to human-related sounds, lung and heart sounds have also been classified using CNNs to help in the diagnosis of pulmonary disorders [45,46] or potential heart diseases [46]. In fact, multiple sounds coming from different sources have been classified with CNNs: from musical instruments [47] to agricultural harvester sounds [48]. For the purpose of this paper, only references related to the classification of urban soundscapes will be considered from now on.

Many papers choose monophonic datasets, such as UrbanSound8K, for implementing classification algorithms using CNNs. Using the UrbanSound8K dataset, Sang et al. achieved accuracy values ranging between 67.41% and 79.06% using different models based on convolutional recurrent neural networks [49] on sub-sampled raw waveforms. Garg et al. worked with the same database using MFCC along with a CNN and a Long Short-Term Memory (LSTM) with test data accuracies ranging from 77% to 82% [50]. Das et al. complemented the CNN with different integrated loss functions achieving even higher accuracies on the same dataset [51]. The same author also tested CNNs and LSTMs combined with up to seven different features, including MFCC and diverse Chroma features with state-of-the-art performance [52]. Continuing with the UrbanSound8K, a comparative approach between CNNs, LSTMs and combinations of these methods was performed in [53] with satisfactory results when they were fed with spectrogram images.

Another monophonic dataset of audio events supported by Real World Computing Partnership was used to test a simple three-layer CNN obtaining better accuracy than most state-of-the-art systems when noise was added [54]. The dataset from the Urban Sound Classification Challenge with 5435 sound events from ten classes was classified using a combination of MFCC and CNN obtaining a 91% accuracy on the test data [55].

### 2.3. Multilabel Classification of Real-World Urban Sounds

Even though literature about multilabel classification of sounds is less abundant, DCASE challenges have raised the number of contributions of the scientific community in recent years. A thorough review of the main publications related to polyphonic sound event detection and classification was provided by Chan et al. [56]. In this review, both non-neural network-based and neural network-based approaches have been included and the datasets and evaluations metrics of the different authors have also been stated. Some of the most prominent contributors to this field are Cakir et al. who classified a dataset with everyday environments with a total duration of 1133 min and a total of 61 different event classes using different Mel-based features and HMM as a classifier. They achieved F1-Scores ranging from 40.7% to 66.9% depending on the binarizing threshold and polyphony level [57]. The same authors compared the performance of multilabel and combined single-label sound event detection and concluded that even though multilabel classification achieves higher accuracy, the combined single-label method scores similar results [58]. More recently, they used Convolutional Recurrent Neural Networks (CRNN) for improving the sound event detection performance on four datasets [59].

Although a significant part of the related literature is covered in the aforementioned review, other more recent contributions include some publications related to the last DCASE challenges [60,61], more focused on indoor domestic sounds, and Vidaña-Vila et al.’s work [62]. In this study, the authors developed a polyphonic sound event classifier for urban data using physical redundancy of sensors. They performed two recording campaigns in Barcelona obtaining almost 300 min of sound data with up to 21 sound classes and implemented a two-stage classifier that benefits from the redundancy sensors to improve the classification performance. The instance average F1-Scores after the first stage ranged from 46% to 75% and the class average F1-Scores ranged from 12% to 39%, depending on which data augmentation method was applied.

## 3. Methodology and Setting

The audio files collected during the 2020 and 2021 *Sons al Balcó* campaigns were labelled following the taxonomy explained in Section 4.1.1. The total number of usable files for each campaign were, then, split into four folds in order to implement a 4-fold cross-validation scheme. On every fold, approximately 75% of data was dedicated to train the machine learning algorithm (including a 10% validation split) and 25% was set apart to subsequently test it. These datasets are highly imbalanced. In fact, some of the sound elements appear in a very limited number of locations. The 4-fold split had to be carefully carried out in order to warrant that all classes were represented both in the train + validation and test subgroups. Therefore, only the correctly annotated sounds from the 30 fine-labelled categories that appeared in at least four locations were considered during the classification process. Moreover, events coming from a single audio recording were placed in either the training or validation sets, to avoid information leakage.

The starting point for the design of the classification algorithm was a previous work by the same authors [37], which was later modified to adapt to the characteristics of the current datasets. In order to extract the features of the studied sound files, a Hamming 30 ms window framing with a 50% overlap (15 ms step) has been used. Increasing this window proved to be detrimental to the performance of the algorithm, especially with those metrics which emphasize a more balanced performance among different sound categories. Bigger time frames are less suitable when there are very short or even impulsive sound events in the dataset such as doors closing or even dogs barking.

For the feature extraction (FE) stage, GTCC have been used. The exact implementation of the GammaTone filter bank is the same as in a previous work by the authors [37] where it is thoroughly explained. GTCC have been chosen because they consistently achieved better results in many more kinds of corpora than other typical FE methods such as MFCC [37]. For each time frame, a vector of 100 coefficients (features) is obtained. Afterwards, the training data are shuffled and normalized before proceeding to the training of the model.

**Figure 1:**
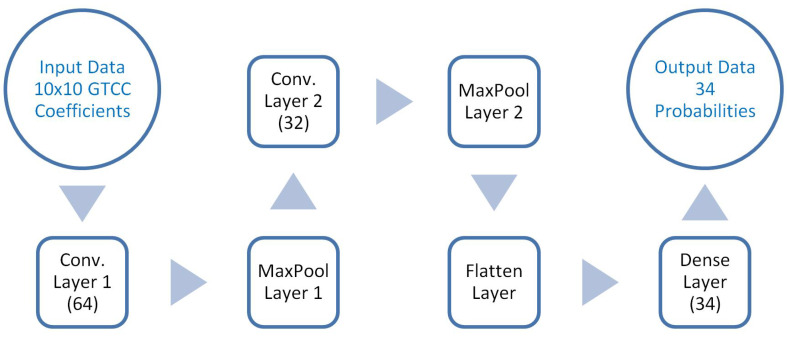
Model of the implemented CNN.

Traditional machine learning algorithms, such as *k*NN successfully used in [37], are not the best suited for complex polyphonic datasets. Instead, this paper proposes a deep learning approach based on a CNN with the setting described in Figure 1.

The implemented CNN consists of two convolutional layers with a 2 × 2 kernel followed by two pooling layers. A total of 64 neurons have been used in the first convolutional layer, while only 32 neurons have been used in the second one. Input data consisting in 100 GTCC coefficients have been previously formatted into a 10 × 10 matrix. The activation function is Rectifier Linear Unit (ReLU). The last two layers are a flattener layer and a dense output layer with 34 neurons, which uses a sigmoid activation function to adapt the model to a multilabel classifier. Moreover, an Adam optimizer with a learning rate (LR) of 0.001 has been used and the loss function was binary_crossentropy.

It is worth noting that increasing the complexity of the network with more layers of bigger kernels or excessively increasing the number of epochs led to an overfitting of the model with higher performance with the training data but a poorer performance overall with the testing subgroup. When classifying only the four most prevalent classes, the number of neurons of the convolutional layers was decreased to 16 and 8, respectively. The simplified classifying algorithm responded better on the reduced datasets.

In order to implement a multilabel classifier, a vector with 34 boolean labels has been assigned to each frame, one for each possible category in the taxonomy, including intermediate labels. A value of 1 is assigned to those categories which are present in the considered frame and a 0 is assigned otherwise.

During the testing stage, a threshold of 0.5 in the computed probabilities has been chosen to consider that a sound element exists in a given frame. Changing this threshold alters the number of true positives and false positives in the detection process. Not all metrics are equally affected by this. If it was important to not miss true positives, it would be advisable to decrease the threshold a little bit, but that would deteriorate the F1-score and other metrics. Given that this study aims to have a broader scope and will use different metrics to assess the results, the 0.5 threshold is the more balanced one.

All the metrics used to evaluate the performance of the classifier were adapted to a polyphonic scenario [63]. Some of the metrics are more suited to the datasets of this project than others. Even though this work presents the accuracy (ACC) results as it is a widely used metric in classification processes, it has to be considered that accuracy is not the best option when dealing with highly imbalanced datasets with many sound classes. The balanced accuracy (BACC) can be an interesting alternative instead as it minimizes the impact of the true negatives (TN) in front of the true positives (TP). Other proposed metrics which may prove to be useful in a wide range of situations are the precision (PRE), the recall or sensitivity (REC), the specificity, the F1-Score and the Error Rate (ER). These metrics enable to evaluate the performance of the proposed model. However, a special focus will be given to the F1-Score and BACC. Not only are they better suited for the current datasets but also are widely used and easy to interpret.

In imbalanced datasets, the classification performance can be very dependent on the specific categories with massive differences within classes. Therefore, it is interesting to not only provide instance averages of the metrics but also class averages (i.e., averages of the metrics of each class). Whereas instance averages consider the different number of samples for each class (i.e., classes with a greater number of samples have more impact on the results), class averages do not take into account the number of samples of each class (i.e., all classes have the same impact on the results).

## 4. Results

This section presents the results of the experimental evaluation of this work. First, Section 4.1 describes and compares the datasets collected during the 2020 and 2021 campaigns. Then, Section 4.2 presents the classification results for both campaigns with all the categories considered. Afterwards, Section 4.3 presents the detailed analysis with only the four most prevalent in the datasets. Subsequently, the effects of the prevalence of each category on performance will be discussed in Section 4.4.

Next, Section 4.5 assesses the effects of the foreground or background placement of a sound during the classification process. Finally, Section 4.6 presents and discusses a viability study using data from a previous campaign to train a model to be used to classify future campaigns. In other words, this last subsection assesses the viability of classifying 2021 data based exclusively on a model trained with 2020 data.

### 4.1. Description of the 2020 and 2021 *Sons al Balcó* datasets

Participants in both *Sons al Balcó* project campaigns were asked to send 30-s audio files recorded with their own smartphones from their balconies. In addition, they were asked to answer a survey describing the soundscape in their locations and their subjective perception of the predominant sounds in each spot. Surveys’ answers added informative metadata to the recordings such as the spatial context or location.

The first part of this section describes the datasets and the taxonomy used to label them. Then, the second part of this section exposes a detailed description of the data collected during the 2020 and 2021 campaigns. Finally, the two datasets are compared.

#### 4.1.1. Taxonomy and General Description

**Figure 2:**
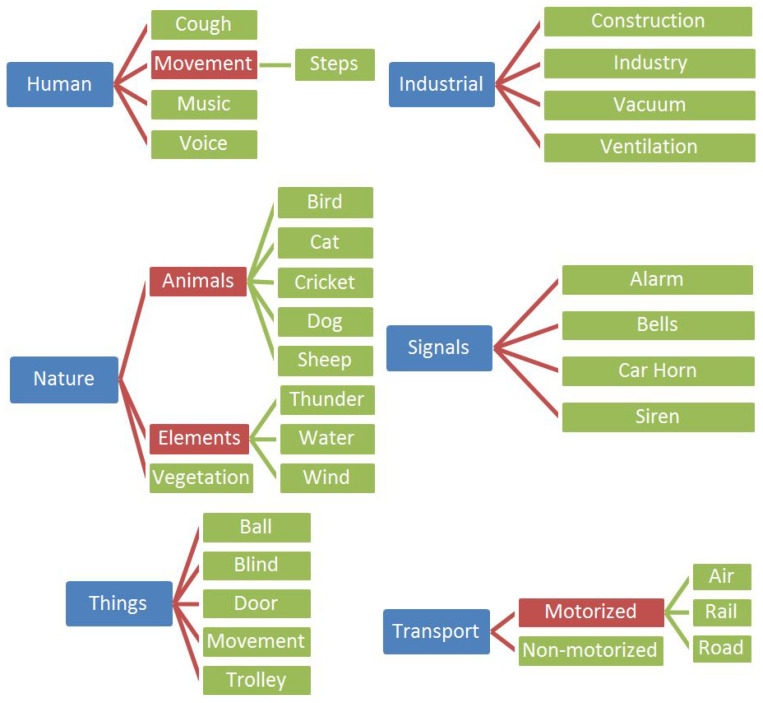
Sound Elements Hierarchical Taxonomy.

During the 2020 campaign, a total of 365 contributions with a total extension of 12,236 s were collected in Catalonia during the campaign. After manually annotating the audio files, 313 locations and 8302.85 s included at least one of the sound categories of the taxonomy in Figure 2.

For the 2021 campaign, 237 participants submitted audio files with a total duration of 7688 s. A total of 236 of these locations and 6951.57 s contained some of the target sound classes. Despite the lower number of contributions in 2021 compared to the 2020 campaign, the total time of meaningful data for sound classification is similar enough.

Both campaigns have been manually labelled using a hierarchical taxonomy. The main categories of sound elements, as shown in Figure 2, are six. First, **Human**, which includes those sounds directly produced by human bodies, but also includes music with human voices. Second, **Industrial**, which focuses on all sounds produced by machinery: from vacuum cleaners to ventilation devices. This category also includes sounds produced by construction sites. Third, **Nature**, which includes sounds produced by animals, vegetation or meteorological elements. Fourth, **Signals**, which includes all sound events related to signalling, such as alarms, bells, car horns or sirens. Then there is the heterogeneous **Things** category, which includes sounds caused by objects (e.g., a door closing or a ball bouncing). Finally, the last category is **Transport**, which includes all sounds produced by motorized and non-motorized transportation.

A hierarchical taxonomy was chosen because it is easily scalable and expandable. For this reason, the main categories described above were derived in different sub-categories. Some of the labels were nonexistent during the 2020 campaign but were added the next year when new sounds appeared (e.g., cough or sheep).

For classification purposes, it is desirable to use specific labels on the lower level of the hierarchy (fine labels). However, some intermediate or higher-level labels have been used during the 2020 and 2021 campaigns when it was not possible to accurately differentiate them from several subcategories, such as in motorized transports, signals or even animals. It is also worth noting that the category *Things/Movement* is quite heterogeneous in its definition since it includes a wide range of different sounds, such as dishes being stacked or a chair being dragged. Heterogeneous sound classes are usually more difficult to automatically classify using machine learning algorithms.

In addition to the sound element description already mentioned, an additional piece of information was added during the labelling process. In particular, the dominion in the soundscape, i.e., the perception of foreground and background placement, was also specified.

**Figure 3:**
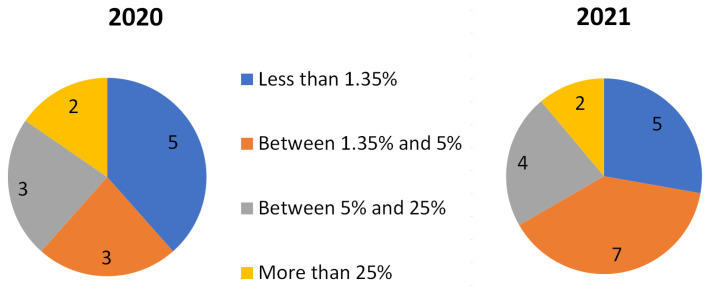
Number of classes by prevalence (% of total time where they appear) and campaign. Classes appearing in less than four locations have been discarded.

From all the different categories defined in the taxonomy, only those classes that appeared in at least four locations were considered for classification. Figure 3 illustrates the number of classes that were present in different locations for each campaign. Thus, only 13 and 18 elements remained for the 2020 and 2021 campaigns, respectively. All 13 of the 2020 sounds appeared also during the 2021 campaign. Table 1 and Table 2 show that the majority of the remaining classes have a low prevalence, not only in terms of the percentage of total time, but also in the percentage of total number of locations. In fact, only 8 and 13 elements for each campaign respectively are to be found in more than 1.35% of the total annotated time length. Furthermore, only four types of sounds are detected in more than 5% of the total time in both campaigns, (i.e., birds, road traffic noise, human voice and wind), as shown in Table 1 and Table 2.

#### 4.1.2. 2020 Lockdown Campaign

Table 1 details the total aggregated time (in seconds) (*Time(S)*) for each sound element during the 2020 campaign along with its percentage prevalence with regards to the total annotated time (*%Time*). It also includes the total amount of available audio clips containing each sound class (*Locations*) and its percentage related to the total number of audio files (*%Locat.*). Only sounds labelled according to the 30 fine categories in the taxonomy that appear in a minimum of four locations are shown. The minimum of four was chosen in order to ensure that every category was represented at least one time in each fold of the 4-fold cross-validation scheme. It also presents the percentage of overlap with other sound elements within each category (*%Overlap*). Occasionally, there are instances where three or more sounds concur. That is depicted in the (*%MultiOverlap*) column.

During 54.65% of the total time, there is a single sound event appearing without any overlap. The distribution of that time among the different classes is shown in column (*%Single*). Finally, the last column (*%Foreground*) contains the percentage of time when a given sound element is labeled as foreground.

**Figure 4:**
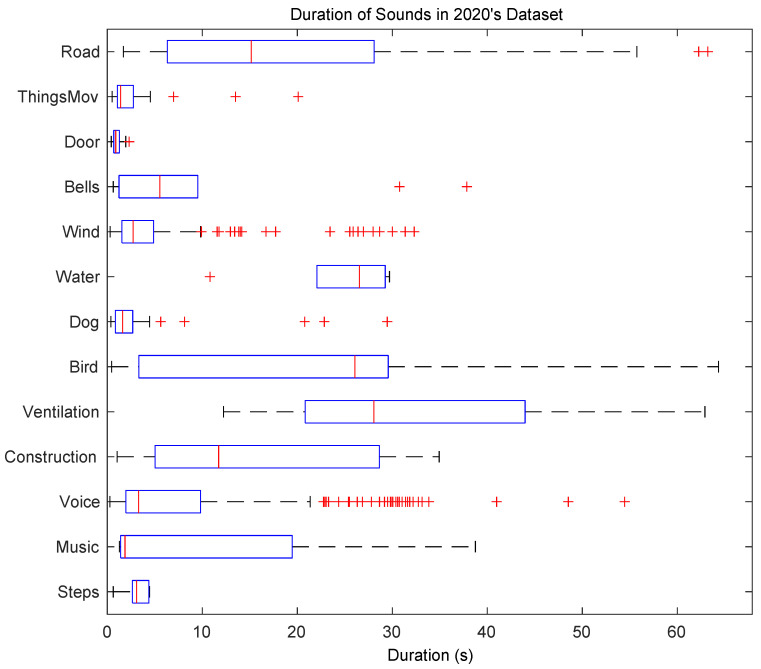
Boxplot of the duration of the different sound elements in the 2020 *Sons al Balcó* campaign. Red crosses indicate the outliers.

From Table 1 it can be concluded that this dataset is highly imbalanced. A total of 2 out of the 13 remaining sound classes, *Bird* and *Road*, appear in more than 30% of the total labelled time and in more than 40% of all available locations. Moreover, six categories, *Steps*, *Music*, *Water*, *Bells*, *Door* and *ThingsMov.*, have a very poor representation with less than 2% prevalence overall. The single and multiple overlapping percentages significantly differ from one element to another. The same happens with the foreground/background placement ratio. Even though in many categories the foreground and background times are approximately balanced, there are also some extreme cases such as *Music*, which are essentially background events, or *Doors*, which have a very poor presence but are always placed foreground. The mean duration of these sound events also presents a wide range of values depending on the type of sound. For a further evaluation of the duration of the events in the campaign, Figure 4 shows a boxplot of the duration of each category in the dataset. Whereas some events have a mean duration shorter than 5 s (e.g., *ThingsMov, Door, Wind, Voice* or *Music*), there are events that last for more than 20 s (e.g., *Road, Water, Birds* or *Ventilation*).

As has been already exposed, this dataset is highly imbalanced with four sound classes that are clearly predominant. On account of that, a separate study based on the detection and classification of these four classes has been conducted and will be further explained in Section 4. These four categories appear in up to 91.43% of the data frames from the test dataset. Furthermore, they appear in more than 80% of the total available fragments and also in more than 80% of the total time available. Therefore, they can be considered the most relevant elements in most of the locations.

#### 4.1.3. 2021 Post-Lockdown Campaign

Table 2 shows the statistics of the 2021 campaign. *Bird* and *Road* are the most prevalent categories with a presence superior to 39% in both cases. Four other classes, *Voice, Wind, Water* and *Ventilation* also appear during more than 5% of the time. On the contrary, up to eight sound elements, *Cough, Steps, Dog, Bells, Car Horn, Door, Rail* and *Non-motorized* appear less than 2% of the total time annotated. Considering the number of locations where each sound class appears, the most wide-spread are *Bird, Road, ThingsMov., Voice* and *Wind*, present in more than 29% of the audio clips. Conversely, *Cough, Steps, Construction, Industry, Ventilation, Bells, Door, Rail* and *Non-motorized* fall short of 5% of the locations.

In 2021, the percentage of overlapping and the foreground-to-background ratio are also significantly different from one sound category to another. In general, the percentage of single and multiple overlaps in 2021 is consistently higher than in 2020 in most categories. There were five new sound elements in 2021 which were not present in 2020’s dataset (marked in italics in Table 2): *Cough, Industry, Car Horn, Rail* and *Non-motorized* transport.

**Figure 5:**
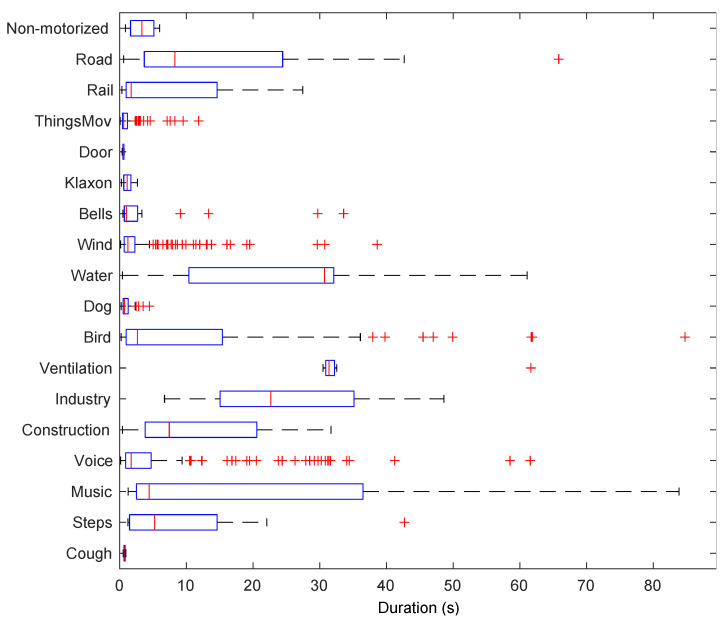
Duration of the different sound elements in the 2021 *Sons al Balcó* campaign.

Figure 5 shows that in 2021, the mean duration of the different categories also had a wide range of values from extremely short elements, such as cough and door, to very long ones, such as music or water, that, when present, normally occupied most of the time in a given location.

The four most prevalent sounds (i.e., birds, road traffic noise, human voice and wind) are clearly the same as in the 2020 campaign. In this case, they appear in 86.43% of the data frames analyzed and in more than 75% of the total available time making these four categories, once more, the most relevant ones.

#### 4.1.4. Comparative of the Two Campaigns

It is interesting to compare both campaigns in greater detail to better understand the possible differences in the classification results. That is especially important given the different conditions and regulations existing during the recording of both datasets.

Comparing Table 1 and Table 2, it can be concluded that, although the dataset from the 2021 campaign is also very imbalanced, the difference of sound prevalence among categories is not as magnified as in 2020. The percentage of time of appearance for each category in the 2020 dataset had a standard deviation of 1313.3 s. This statistic falls to 932.64 s in 2021 as seen in Table 3. The two more prevalent categories (*Birds* and *Road*) are also more balanced in 2021. While in 2020 there was a significant 22.15% difference in the percentage of total time between both of them, the gap falls to 7.59% in 2021. The number of categories which appear more than 2% of the time increases from 7 in 2020 to 10 in 2021. Some of the differences between both datasets are accidental, such as the higher percentage of locations where it was raining in 2021 compared to 2020. However, part of the higher range of existing sounds in 2021 can be explained because the 2020 campaign was conducted during the COVID-19 lockdown while in 2021 most of the mobility and activity were back to normal [64].

**Figure 6:**
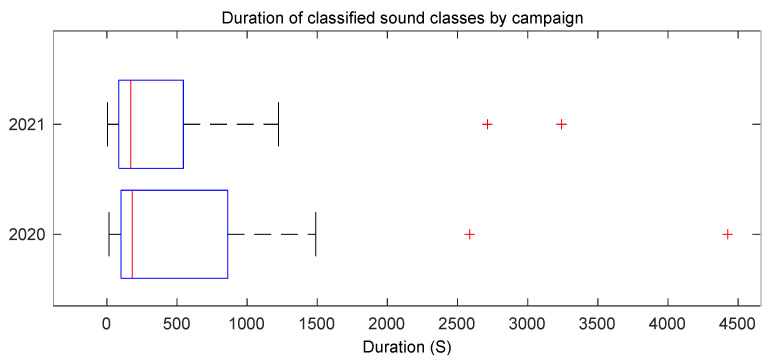
Comparative between 2020 and 2021’s aggregated duration of the sound categories.

Table 3 summarizes the most relevant differences in the statistical composition of both datasets. As it was expected, 2021 offers a wider range of sound categories. Also, the number of sound events per location is more than doubled compared to 2020. The mean duration of the sound events drops to almost a half, from 12.51 s to 6.57 s. That is partly due to a decrease in the number of short events caused by a decline in human activity during the lockdown period of 2020. There is also a noticeable difference when comparing the aggregated duration of the sound categories in both campaigns as shown in Figure 6.

Other relevant changes from one campaign to the other include a stark boost in the percentage of time with overlapping of two or more sound categories from roughly 45% in 2020 to over 60% in 2021. Also, the percentage with three or more sound categories appearing simultaneously expands from 8.1% to 10.53% (Table 3). Finally, a slight increase of just over 5% in the foreground-to-background ratio is perceived in 2021.

The relevance of the different classes changed from 2020 to 2021. During the lockdown, the majority of the sounds were nature-related. In fact, paying attention to the coarse tags in the taxonomy, 50.76% of the annotated sounds during the COVID-19 pandemic belonged to nature. This percentage shifted in 2021 where only 43.11% of the sound events were caused by animals or elements from nature. Signals and things more than doubled their presence in 2021. Sound events tagged in the human category also increased. Transport audio events raised their presence by more than 3%, which is consistent with the end of the mobility restrictions. By contrast, industrial-related sounds showed little variation between both campaigns as construction works were already resumed during the 2020 recordings.

### 4.2. General Results for the 2020 and 2021 Campaigns

Table 4 displays the classification metrics for *Sons al Balcó* datasets and the classification algorithm explained in the previous section. The first column (2020) shows the main results of the automatic classification process of the sounds when only the acoustic data of the 2020 campaign is used. Next, column 2021) presents the corresponding metrics when using only the acoustic data of the 2021 campaign. Finally, the last column (2020 + 2021), shows the performance of the algorithm when both campaigns are aggregated. All the metrics result from a 4-fold cross-validation scheme as explained in Section 3.

Focusing on the metrics for the 2020 campaign, there is a significant difference between the instance average-based metrics and the class average ones, as was expected. The main reason is that there is a vast variation in the classification performance among classes. While from an instance point of view, a balanced accuracy of 73.05% and an F1-Score of 54.75% are achieved, these values drop steeply to a BACC of 54.91% and, especially, an F1-Score of 16.3% when the class average is considered.

As stated in Section 4.1.4, the composition of the dataset corresponding to the 2021 campaign is more complex. It has more variety of sounds, it doubles the number of sound events per location, the events mean duration is approximately half that of 2020, it almost triples the number of category shifts per minute, and it has over 30% increase in the overlap and multiple overlap percentages.

The instance average of all the metrics, except for the accuracy and specificity, is slightly inferior in 2021 when compared to 2020, as seen in Table 4. For example, the F1-Score drops from 54.75% to 51.37%. Conversely, most of the metrics in the class average improve thanks to the better classification performance of some less frequent sounds, such as *rail*.

### 4.3. Results for the Four Most Prevalent Sounds in the 2020 and 2021 Datasets

As seen in Table 1 and Table 2, there are four classes that stand out for their higher presence. In this subsection the results come from a model specifically trained to classify these four elements.

Focusing on the 2020 campaign, in general, differences between classes are clearly reduced, as shown in Table 5. A direct consequence is that class average metrics and instance average metrics are significantly more similar in this subset of sound elements. Almost all metrics give better results compared to those in Section 4.2, except for accuracy and specificity, whose decline was expected with the reduction of studied categories from 13 to 4. The higher improvement can be detected in the class average metrics, as the number of True Negative events has diminished with the narrowing of categories. However, instance average metrics also present a significant increase.

Balanced accuracies exceed 64% in three out of four classes. Focusing on the instance averages, the BACC is close to 75% and the F1-Score surpasses 65%. In addition, the averages of all metrics overcome 50%, except for the class average recall. When comparing the four categories, the specificity of birds stands out for being much lower. This is due to the oversized number of false positives (FP) in this category, which is the most prevalent across the dataset with a presence nearing the double of the subsequent category.

As for 2021, when the model is trained to identify only the four predominant categories, the performance improves in a similar way as in 2020. As seen in Table 5, differences among classes are reduced, even more than in 2020. However, birds and road traffic continue to prevail as the better-classified elements. Class average metrics and instance average metrics are closer.

Balanced accuracies exceed 69% in three of the four classes. F1-score fluctuates between 19.45% for the voice and 77.75% for the birds, with three classes above 50%. Specificity is more uniform throughout the four categories, but it is still lower for the birds.

### 4.4. Effects of the Prevalence of the Classes on Its Classification Performance

The low values from the class average metrics are better explained looking at the specific results for each kind of sound. It is convenient to differentiate between three big groupings depending on the percentage of prevalence: the more prevalent categories which appear more than 25% of the time, the classes with intermediate prevalence from 5% to 25% and, finally, the classes with lower than 5% of prevalence.

**Figure 7:**
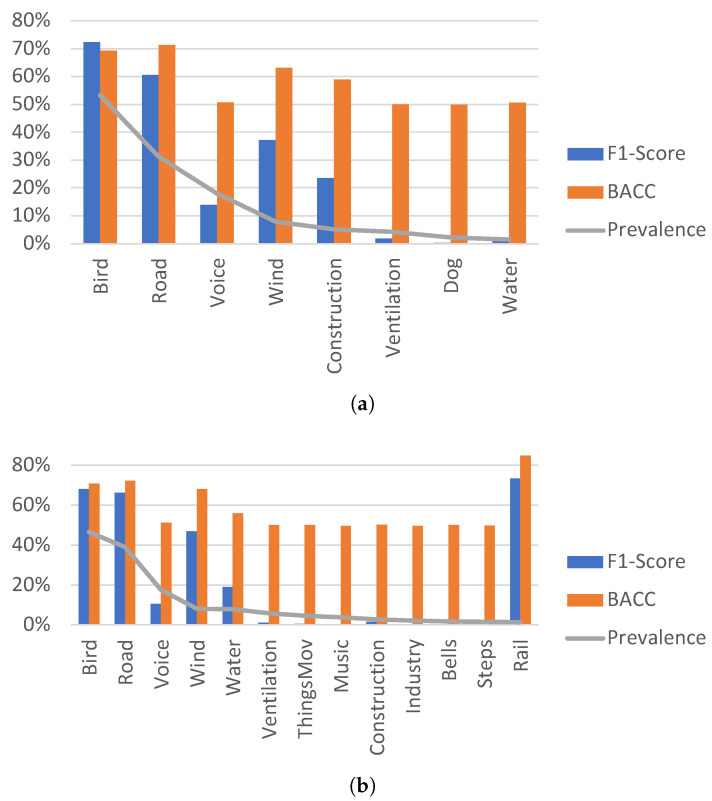
F1-Score and BACC for the categories that have a presence higher than 1.35% of the total time during (**a**) 2020 and (**b**) 2021.

Figure 7a shows the F1-Score and BACC for the 2020 classes with a prevalence greater than 1.35%. The classes with lower prevalence are not correctly classified. The two most prevalent sound categories, *bird* and *road traffic noise*, with a presence of over 25%, have an F1-Score greater than 60%, an error rate less than 0.75 and a BACC close to 70%.

The three following classes (sorted by percentage of time of appearance) are *voice, wind* and *construction*. They appear between 5.17% and 17.93% of the total time in the dataset, belonging to the second grouping. All of them have a BACC higher than 50% and F1-Score higher than 13%. In fact, *wind* and *construction* perform even better with a BACC exceeding 59% and an F1-Score of more than 23%. On the contrary, *voice* performs worse even though its presence is higher than *wind* and *construction*. This can be explained because the foreground to background ratio in the voice is low (most of the time the voice is placed in background) while wind and construction are normally placed in foreground. In fact, the sounds that are predominantly placed in background: *voice, ventilation* and *water*, obtain worse results than those with similar presence but with a dominant foreground placement. Therefore, the foreground-to-background ratio seems to be relevant in the classification performance. This issue will be further discussed in Section 4.5.

The categories with less prevalence than the five aforementioned have very poor classification results. Their balanced accuracies are close to 50%, which makes them unreliable, and their F1-Scores fluctuate between 0% and 2%.

Figure 7b presents the detailed F1-Score and BACC for the thirteen sound categories that appeared at least 1.35% of the time during the 2021 campaign. As in 2020, there is a correlation between the prevalence of the sound and its F1-Score with a couple of notable exceptions.

The two more prevalent classes, *bird* and *road traffic noise*, which belong to the first grouping, have F1-Scores that exceed 65%. The next three classes, which appear a minimum of 7.5% of the total time, have F1-Scores fluctuating between 10.55% for the voice and almost 47% for the wind. The other class with more than 5% of presence is ventilation, which has a F1-Score barely over 1%. Voice and ventilation have a significantly worse foreground-to-background ratio than wind and water.

The sound categories with less than 5% of presence have very poor performances except for *rail* and, to a smaller extent, *construction*. Rail proves to be a sound especially easy to detect and classify with an F1-Score close to 75% with a low prevalence of only 1.36% of the time. However, it must be highlighted that it is always placed in the foreground and its overlap with other sounds is inferior to 50%. Construction is another category with similar features, with a foreground placement over 95% and an overlapping under 50%. Again, foreground-to-background ratio continues to play a role in improving classification performance.

### 4.5. Effects of the Foreground and Background Placement in the Classification Process

**Figure 8:**
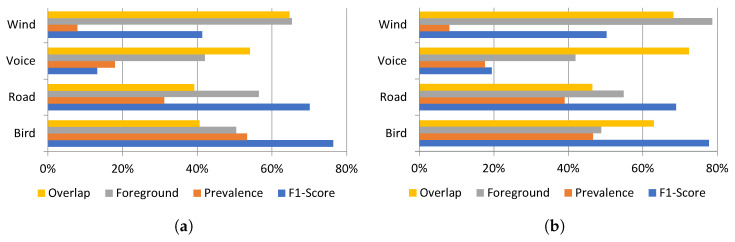
Comparative of the F1-Score of the 4 most prevalent classes related to their prevalence, overlapping and foreground to background ratio in (**a**) 2020 and (**b**) 2021.

There are many factors that can influence the disparity in the classification performance among different categories. In Figure 8, three features (prevalence, foreground placement and overlapping with other sounds) of the four most prevalent sound classes are studied in order to find possible correlations with the performance and, more specifically, with the F1-Score. Effectively, there is a certain relationship between the percentage of prevalence of a sound in the dataset and its F1-Score, as was already stated in Section 4.4. Presences over 20% produce high F1-Scores with independence of other features.

Another element of the composition of the dataset that proves to be relevant is the foreground/background ratio. In general, higher foreground-to-background ratios lead to higher F1-scores. Thus, *wind* has a better performance than *voice* even though its presence is lower. Also, *road traffic noise*’s lower presence related to *birds* is partly compensated with its higher foreground-to-background ratio achieving a similar F1-Score. In contrast, the relevance of the percentage of time with overlapping cannot be stated with the analyzed data.

Figure 8b shows the same correlation for 2021 between sound presence and F1-Score that had already manifested in 2020 (Figure 8a), reinforcing the relevance of the foreground-to-background ratio as a booster of the performance in the classifying process.

**Figure 9:**
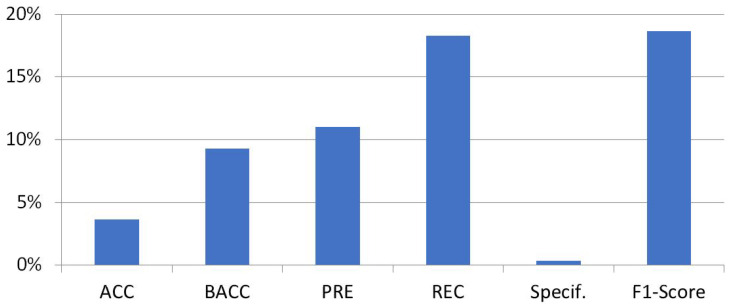
Mean improvement for 2020 and 2021 in the classification performance of foreground sounds compared to background sounds.

To complement these observations, a separate computing of the instance average metrics has been made to compare the testing results of foreground sounds against background sounds in both campaigns. Effectively, a significant difference was spotted. As can be seen in Figure 9, the performance of foreground sounds surpasses the results of background sounds in every metric, with improvements ranging from 0.36% to almost 19%. The metrics that show the most difference are the recall and the F1-Score.

### 4.6. Testing a Trained Model on a Different Campaign

One of the goals of this project is to study the viability of training a model with available data from past campaigns with the purpose of classifying future campaigns. To achieve this, another experiment was conducted. The model has been trained exclusively with all the data available for the 2020 campaign and, subsequently, it was tested with all the data collected during 2021.

The obtained results are similar enough to those compiled in the 2021 column on Table 4. Focusing on the F1-Score, its instance average only dropped 1.56%, from 51.37% to 49.81% when the model was trained with 2020 data. Its class average fell slightly more, a 4.6%. Regarding the balanced accuracy, the decline is even softer, of only 1.46% and 2.13% for the instance and class averages. Comparing the performance in the classification of the four most prevalent sounds, metrics drops are even smaller, fluctuating between 1.07% and 2.47%.

It has to be noted that both datasets have significant differences to the point where some categories do not even exist in one of the campaigns. It is to be expected that future campaigns’ datasets composition will be more similar to 2021 now that a post-pandemic scenario is emerging.

## 5. Discussion

Section 5.1 analyses and evaluates the performance of the acoustic event classification algorithm according to the different metrics and compares the results with other state-of-the-art propositions. Later, Section 5.2 compares the results of both campaigns along with the results of aggregating the data of both datasets for the training and testing.

### 5.1. Performance of the Acoustic Event Classification

As seen in Table 4 and Table 5, the best metrics are the accuracy and the specificity, which both offer excellent results exceeding 90% when all categories are considered. This was expected given the imbalanced characteristics of the dataset. Additionally, analysing the system performance, it offers better precision rates than recall and much better specificity than the other two metrics. Having a better precision rate than a recall rate means that the system is more confident in predicting true values (most of the positive predictions are actually correct, so the confusion between positive classes is low) than classifying correctly all the positive events (the system may predict as negative a class that is actually present in the audio fragment). The specificity gives information about how many of the negative predicted events are actually negative. In this specific problem, the elevated specificity value is due to the high amount of negative samples present in the dataset, as in a multiclass approach, a sample that contains data from a single category will be considered as a positive event for that category and a negative event for the rest of the categories. This high value indicates that the system correctly classifies as negative most of the events that are actually negative.

Focusing on F1-Score, this work proposal achieves micro F1-Scores of 51.37% to 54.75% when all the categories on the datasets are considered. These values rise to 65.22% and 66.2% when classifying only the most prevalent noise sources, i.e., those with higher than 5% presence. Furthermore, there is a remarkable contrast in the classification performance (19% variation on the F1-Score) of foreground sounds and background sounds. Similarly, the macro F1-Scores of 16.26% and 16.3% with the full dataset increase to 50.23% and 54.09% in the subset of predominant noise sources.

The performance of automatic sound classification in polyphonic environments is affected by the composition of the dataset. Features such as the number and typology of classes or the number of instances for each category have an impact. Most of the recent literature on polyphonic sound events classification focuses on indoor sounds and balanced datasets with fewer categories making it more difficult to use as a baseline. With that assumption, there are some recent works that are rated with the same metric as this one (event based F1-Score) that can be useful as a reference. A recent previous work from one of the authors [62] achieved a micro F1-Score of 46% and a macro F1-Score of 12% with a similar dataset of 21 classes of outdoor urban sounds prior to data augmentation. The top ranked work in the DCASE2020 Challenge Task 4 [65] (DCASE2022 chose a different metric to assess the performance) scored 41.7% in the event based F1-Score before data augmentation using a dataset of ten classes of indoor sounds. Even though the results of the proposed system are worse when the full dataset is considered, the F1-Score with the subset of most prevalent sounds is significantly higher (8.53 to 12.39%) than the winner of the DCASE2020 Challenge. These results are consistent with the complexity of the dataset and the number of classes considered.

### 5.2. Comparative and Aggregation of 2020 and 2021 Campaigns

The 2020 campaign was conducted during the lockdown caused by the COVID-19 pandemic. Thus, the soundscape of many locations was affected. The 2021 campaign, on the contrary, took place during a back-to-normal post-lockdown situation. It is interesting to compare the classifying results in both situations.

Looking at the results in Table 4, there are not significant differences between both campaigns. However, some disparities are spotted. On the one hand, comparing the instance average metrics, almost all of them improve the results during the 2020 as was to be expected because the dataset was simpler. On the other hand, most of the class average metrics perform better in 2021, apart from a minimum decrease of the F1-Score, because the 2021 dataset is slightly better balanced.

That being said, differences among campaigns are minimal. In fact, most metrics present less than 1.5% variation from 2020 to 2021. Given the extremely different context in which the data was collected, the similar performance of the algorithm betokens its reliability in analysing different urban soundscapes.

It is also interesting to study the effects of aggregating both campaigns, which would double the amount of available data for training the model. The results are shown in the last column of Table 4. Half of the metrics based on instance average, (i.e., F1-Score, BACC, and Recall), achieve performances in between both campaigns. Conversely, instance average accuracy, precision and specificity and most of the class average based metrics, with the exception of Recall, improve their results when both datasets are combined.

When dealing with a highly imbalanced dataset, increasing the number of categories and the amount of data raises the number of true negatives (TN). As a result, accuracy and specificity are improved. The improvement in most of the class average metrics takes place because the expanded dataset gives more representativeness to some of the less prevalent sound categories.

## 6. Conclusions

Two polyphonic multi-sourced annotated datasets originated from a citizen science project have been used. The 2021 dataset composition is substantially different from the prior year. While 2020 data was recorded during a lockdown with heavy restrictions on human activities and mobility, 2021 corresponds to a regularized year with standard intensity and variety of activity. Even so, the performance results for automatic classification of sounds are not significantly different in both campaigns, which is an indicator of the robustness of the algorithm.

Having highly imbalanced datasets with abundant categories, the metrics that are more suited to evaluate the classification performance are F1-Score, balanced accuracy, sensibility and error rate instead of unbalanced accuracy or specificity. Results are considerably better when the classification performance is computed globally at instance level rather than when individual performances per class are averaged.

Not all categories are equally classified. Some features of the dataset composition affect the performance. The percentage of prevalence of a specific category within the dataset plays a decisive role. There is a positive correlation between prevalence and several metrics. F1-Score is especially improved in those sounds that appear more often. Sound elements that appear less than 5% of the total time usually are not properly classified by the algorithm. On the other hand, those with more than 20% of presence achieve satisfactory results. Those with percentages of presence in between have modest performances. Another feature that weighs positively on the performance is the foreground-to-background ratio of each sound category. This feature alone, or combined with low overlapping rates, usually translates to improvements in the F1-Score.

The system improves extensively when it is focused on detecting and classifying a reduced set of predominant categories, more specifically, those with a presence of over 5% of the total time in both datasets.

In order to improve the performance, the authors propose to increase the data used for training the model. It will have a positive impact on most of the class average metrics as proved in Section 5.2. Data with a polyphonic composition comparable to the datasets already used are the best suited for doing so, (e.g., data collected in future campaigns) or even synthetically augmented data.

The removal of pooling layers from the neural network model can have a positive impact on some metrics when working with all categories. Specifically, class average F1-Score and BACC are slightly improved since some low-presence categories, such as *music* and *dog*, are better classified. However, this improvement comes at the cost of a decline in other metrics, such as the error rate and some instance-level metrics, because categories such as *bird* have worse results. All in all, variations are not significant in either case. Adding more complexity to the neural network with more neurons or more layers is counterproductive as the system tends to overfit.

Data collected during one specific campaign can be used to detect and classify sounds from future campaigns with a similar, even if slightly lower, performance than when data from the same campaign are used to train and test the model. This highlights the benefit of aggregating available data from past campaigns to classify future ones.

Even though the system could be applied to any polyphonic dataset, it is best suited to detect the predominant sound sources in an urban soundscape, especially when they are located foreground. It has been tested on real-life data obtained from a citizen science project, with state-of-the-art performance in detecting prevalent sound categories, such as road traffic noise or birds, which are some of the most relevant noise sources when assessing the quality of an urban soundscape. Thus, the results are encouraging for the purpose for which they were designed.

Future work will be focused, on the one hand, on improving the machine learning algorithms results and enabling the possibility of computing real-time detection for the evaluation of any video uploaded during a recording campaign, and on the other hand, in providing a merged analysis of data, considering both the results of the questionnaires when asking the perception about positive and negative sounds, with the outputs of the acoustic event detection algorithm.

## Figures and Tables

**Table 1:** Dataset’s internal statistics for the 2020 campaign (only fine labels appearing in 4 locations or more).

Event	Time (s)	Locations	%Time	%Locat.	%Overlap	%MultiOverlap	%Single	%Foreground
Steps	18.26	6	0.16%	1.92%	3.43%	0%	0.16%	51.64%
Music	111.3	9	1.34%	2.88%	45.76%	20.09%	0.55%	9.24%
Voice	1488.9	103	17.93%	32.91%	54.07%	7.99%	6.26%	42.03%
Construction	429.3	23	5.17%	7.35%	53.41%	23.17%	1.83%	55.97%
Ventilation	350.24	10	4.22%	3.19%	31.19%	0%	2.21%	28.94%
Bird	4425.8	177	53.3%	56.55%	40.58%	6.37%	24.06%	50.42%
Dog	181.59	20	2.19%	6.39%	59.71%	3.65%	0.67%	68.56%
Water	121.97	5	1.47%	1.6%	99.34%	23.86%	0.01%	21.17%
Wind	653.01	65	7.86%	20.77%	64.67%	18.16%	2.11%	65.34%
Bells	100.69	10	1.21%	3.19%	80.43%	3.82%	0.18%	58.57%
Door	14.89	11	0.18%	3.51%	73.34%	0%	0.04%	100%
ThingsMov.	99.81	27	1.2%	8.63%	39.35%	6.2%	0.55%	62.14%
Road	2586.8	128	31.16%	40.89%	39.17%	6.58%	14.4%	56.51%

**Table 2:** Dataset’s internal statistics for the 2021 campaign (only fine labels appearing in 4 locations or more). The elements that are marked in italics were not present in the 2020 campaign.

Elements	Time(s)	Locations	%Time	%Locat.	%Overlap	%MultiOverlap	%Single	%Foreground
*Cough*	*4.2*	*4*	*0.06%*	*1.69%*	*85.62%*	*21.67%*	*0.01%*	*100%*
Steps	107.48	10	1.55%	4.24%	78.97%	28.31%	0.22%	55.42%
Music	264.44	12	3.80%	5.08%	91.69%	12.68%	0.21%	94.61%
Voice	1223.6	91	17.60%	38.56%	72.36%	17.02%	3.29%	41.96%
Construction	190.85	9	2.75%	3.81%	42.10%	2.08%	1.07%	95.28%
*Industry*	*150.79*	*5*	*2.17%*	*2.12%*	*29.92%*	*1.07%*	*1.03%*	*66.66%*
Ventilation	405.25	11	5.83%	4.66%	63.38%	11.46%	1.44%	15.63%
Bird	3241	140	46.62%	59.32%	62.94%	9.64%	11.68%	48.84%
Dog	85.36	22	1.23%	9.32%	85.51%	53.23%	0.12%	80.76%
Water	545.79	18	7.85%	7.63%	59.57%	12.16%	2.15%	78.86%
Wind	559.96	69	8.06%	29.24%	68.24%	12.44%	1.73%	78.61%
Bells	117.83	10	1.69%	4.24%	84.66%	3.39%	0.18%	67.94%
*CarHorn*	*20.4*	*12*	*0.29%*	*5.08%*	*87.04%*	*17.35%*	*0.03%*	*84.28%*
Door	4.31	8	0.06%	3.39%	88.20%	27.36%	0.00%	100.00%
ThingsMov.	319.47	115	4.60%	48.73%	81.17%	20.96%	0.58%	93.88%
*Rail*	*94.68*	*7*	*1.36%*	*2.97%*	*42.32%*	*3.04%*	*0.53%*	*100.00%*
Road	2713.2	124	39.03%	52.54%	46.49%	5.96%	14.11%	54.86%
*Non-motor*	*13.49*	*4*	*0.19%*	*1.69%*	*33.81%*	*0.28%*	*0.09%*	*68.37%*

**Table 3:** 2020 and 2021 campaign dataset’s comparative.

Dataset Feature	2020	2021
Fine-labelled sound classes present	23	27
Fine-labelled sound classes (>4 locations)	13	18
Events per location	2.86	6.62
Event mean duration (s)	12.51	6.57
Event duration’s Std. Dev. (s)	12.93	11.17
Sound categories mean duration (s)	814.04	559
Sound category duration’s Std. Dev. (s)	1313.3	932.64
Sound changes per minute	8.77	21.14
%Overlap	45.24%	60.68%
%Multiple Overlap	8.10%	10.53%
%Foreground Placement	50.96%	56.54%

**Table 4:** Summary of Metrics assessing the classification performance for the 2020 campaign, the 2021 campaign and both campaigns aggregated.

	Metric	2020	2021	2020 + 2021
	ACC	91.84%	93.19%	94.1%
	BACC	73.05%	71.05%	72.28%
	PRE	61.83%	60.57%	65.09%
Instance Average	REC/Sensibility	49.46%	44.66%	46.58%
	Specificity	96.63%	97.45%	97.98%
	F1-Score	54.75%	51.37%	54.21%
	ER	0.60	0.64	0.60
	ACC	91.84%	93.19%	94.1%
	BACC	54.91%	55.73%	55.85%
	PRE	20.47%	21.39%	21.5%
Class Average	REC/Sensibility	14.86%	15.07%	14.71%
	Specificity	94.97%	96.39%	96.99%
	F1-Score	16.3%	16.26%	16.4%
	ER	1.10	1.10	1.04

**Table 5:** Summary of Metrics assessing the classification performance for the four most prevalent sounds during the 2020 and 2021 campaigns.

**2020 Campaign**							
	**ACC**	**BACC**	**PRE**	**REC**	**Specif.**	**F1Score**	**ER**
Voice	76.02%	51%	37.97%	8.98%	93.01%	13.18%	1.23
Bird	71.68%	70.31%	75.33%	77.97%	62.65%	76.36%	0.47
Wind	92.17%	64.76%	66.70%	31.37%	98.15%	41.33%	0.88
Road	79.65%	76.69%	73.85%	67.66%	85.72%	70.07%	0.58
Class Avg.	79.88%	65.69%	63.46%	46.5%	84.88%	50.23%	0.79
Instance Avg.	79.88%	74.67%	70.37%	60.82%	88.52%	65.22%	0.47
**2021 Campaign**							
	**ACC**	**BACC**	**PRE**	**REC**	**Specif.**	**F1Score**	**ER**
Voice	77.82%	53.95%	33.45%	14.13%	93.77%	19.45%	1.12
Bird	75.18%	75%	74.27%	81.97%	68.04%	77.75%	0.47
Wind	92.43%	69.66%	64.18%	41.64%	97.67%	50.3%	0.81
Road	73.96%	73.37%	75.23%	64.28%	82.45%	68.88%	0.58
Class Avg.	79.85%	67.99%	61.78%	50.51%	85.48%	54.09%	0.74
Instance Avg.	79.85%	75.1%	71.37%	61.91%	88.29%	66.2%	0.47

## Data Availability

Not applicable.

## Abbreviations

The following abbreviations are used in this manuscript:

ACC	Accuracy
BACC	Balanced Accuracy
BiLSTM	Bidirectional Long Short-Term Memory
CNN	Convolutional Neural Networks
CRNN	Convolutional Recurrent Neural Networks
DCASE	Detection and Classification of Acoustic Scenes and Events
ER	Error Rate
FE	Feature Extraction
FLD	Fisher’s Linear Discriminant
FN	False Negatives
FP	False Positives
GMM	Gaussian Mixture Model
GTCC	GammaTone Cepstrum Coefficients
HMM	Hidden Markov Models
*k*NN	*k*-Nearest Neighbor
LPCC	Linear Prediction Cepstrum Coefficients
LR	Learning Rate
LSTM	Long Short-Term Memory
MFCC	Mel Frequency Cepstrum Coefficients
NB-ACF	Narrow Band Auto-Correlation Features
PRE	Precision
REC	Recall
ReLU	Rectifier Linear Unit
SVM	Support Vector Machines
TN	True Negatives
TP	True Positives
WHO	World Health Organisation
